# The Interplay of Race, Ethnicity, and Language in Caregiver Health: Insights from the National Social Life, Health, and Aging Project

**DOI:** 10.21203/rs.3.rs-4178612/v1

**Published:** 2024-04-04

**Authors:** Lissette M. Piedra, Selena Zhong, Melissa J. K. Howe, Ellen Compernolle, James Iveniuk

**Affiliations:** University of Illinois Urbana-Champaign; National Opinion Research Center; National Opinion Research Center; University of Illinois Urbana-Champaign; National Opinion Research Center

## Abstract

**Background:**

Recent socio-demographic shifts in the United States have underscored the growing importance of informal caregiving and raised concerns about caregivers’ health and well-being. This study aims to deepen our understanding of the health dimensions of caregivers, considering their diverse backgrounds.

**Objective:**

To examine five key health dimensions (physical, cognitive, mental, social, and sexual health) of caregivers, and to identify potential disparities based on ethnoracial and linguistic differences.

**Methods:**

Using data from the National Social Life, Health, and Aging Project (NSHAP), this study explores the interconnections among the specified health dimensions of caregivers and their ethnoracial (Black, Hispanic, White, and others) and linguistic (Spanish, English) backgrounds, in addition to their social networks (N=1,309). Regression analysis was employed to discern the patterns and associations.

**Results:**

The findings indicate that White caregivers generally report better physical, cognitive, and social health compared to their Black and Hispanic counterparts, but exhibit less favorable outcomes in sexual health than Hispanic caregivers. Spanish-speaking caregivers, while having lower cognitive and self-rated mental health than English-speaking caregivers, show stronger social health and greater relationship satisfaction. Notably, these correlations persist irrespective of the size of social networks, pointing to intrinsic links with health outcomes.

**Conclusion:**

The study underscores the necessity of a comprehensive health evaluation for caregivers, acknowledging the intricate interplay between their health and various socio-demographic factors. It advocates for the development of targeted policies and interventions that address the complex health needs of caregivers, with an emphasis on their ethnoracial and linguistic contexts and social environments.

## Introduction

Over one in five Americans engage in family caregiving, and 37.1 million people in the U.S. provide unpaid eldercare according to the National Alliance for Caregiving (NAC), the AARP, and the U.S. Bureau of Labor Statistics (2023) (1–3). Such widespread caregiving signifies a profound societal shift, emphasizing the vital role of caregivers as life expectancies increase. This societal shift underscores the need to focus on the well-being of caregivers by thoroughly exploring their experiences, and the impact of the care they provide on their personal health (1, 2, 4).

The current study aims to deepen our understanding of these issues in three key areas by: 1) Investigating caregivers’ health across five dimensions, including physical, mental, cognitive, social and sexual health, using the “linked health” concept; 2) Exploring the connection between caregivers’ ethnoracial and linguistic backgrounds and their comprehensive health profiles; and 3) Assessing the influence of social networks on caregiver health (4, 5). For this analysis, we use data from the National Social Life, Health, and Aging Project (NSHAP), a nationally representative, longitudinal survey of older adults and their cohabiting spouses and romantic partners who live in communities across the United States (6).

### Linked health

The concept of “linked lives” that is integral to life course theory, posits that our lives are deeply intertwined with, and influenced by, our social environments. This concept, underscored by researchers like Bengtson (2016) and Carr (2018), suggests that individual experiences, such as facing a disability or becoming a caregiver, not only affect the person directly involved but also have significant repercussions on the well-being of their close relations (3, 7). This notion extends to the concept of “linked health,” which implies that a change in one aspect of a person’s health can impact others, triggering a chain reaction that affects their overall well-being. Studies, including those using NSHAP by Waite and Das (2010) and Waite et al. (2021), have shown strong, often bidirectional, links between social health and other health dimensions—be it physiological, functional, psychological, or cognitive (8, 9). For example, sexual health, often neglected in discussions on aging, plays a crucial role in older adults’ overall well-being. The frequency and quality of sexual activity have positive effects on health, contributing to improved cardiovascular health, reduced psychological distress, and enhanced happiness and subjective well-being (10–12). Research shows that partnered sexual activity persists despite cognitive impairment, suggesting that such activities, which are inherently social, are more common than solitary behaviors like masturbation in individuals with cognitive limitations (11). This interconnectedness of health dimensions provides a compelling reason to examine the multifaceted health profiles of informal caregivers. Given the substantial evidence highlighting experiential differences along demographic lines, it is particularly crucial to explore the varied health profiles of informal caregivers across different ethnoracial and linguistic backgrounds.

### Caregiver diversities

Caregivers are not a monolithic group; their experiences differ widely by ethnoracial background, socioeconomic status, and caregiving responsibilities, along with exposure to cumulative disadvantage (13–16). According to the National Association of Chronic Disease Directors and the Centers for Disease Control (2018), about 22.3% of adults have recently provided care to a friend or family member, with women more likely to be caregivers than men (25.4% vs. 18.9%). Caregivers are diverse in terms of race, with 23% identified as White, 24.3% as Black, and 17.9% as Hispanic adults (5). Many provide substantial amounts of care, with 31.3% contributing 20 or more hours weekly and 53.8% doing so for over 24 months (5). Furthermore, 10.4% care for someone with dementia or cognitive impairment.

However illuminating, such statistics do not fully reveal the inequities faced by ethnoracial minority caregivers, who often experience higher depression levels and spend more time caregiving with fewer external supports (17–21). For example, Black caregivers face significant disparities in caregiving intensity, economic hardship, and service accessibility (21, 22). Language barriers further complicate caregiving, especially for those with limited English proficiency, exacerbating access issues to needed health and respite services and creating an overreliance on bilingual caregivers (19, 20, 23–28).

Despite facing higher stress levels, caregivers from minoritized ethnoracial groups often report positive caregiving gains, such as fulfillment and personal growth, more so than their White counterparts. This includes Hispanic and Black caregivers reporting greater appreciation for life and a positive attitude towards caregiving (29–32). This complex mix of challenges and rewards influencing caregiver health necessitates a nuanced assessment that acknowledges the roles of social context and relationships. Health disparities across ethnoracial and linguistic groups highlight the need to address this interplay, especially given the significant impact of racial disparities and social inequities on caregiver well-being. They also raise the question of how different aspects of health, in particular the social resources that contribute to social health, are linked with cognitive, physical, sexual, and mental health; within this framework, social resources are defined as the relationships that support and strain caregivers (9). To address these issues, we present a multifaceted assessment of health among ethnoracial and linguistically diverse caregivers that focuses on social context and emphasizes relationships.

### The influence of social networks on health

Research consistently shows a strong link between the quality of an individual’s social connections and positive health outcomes, underscoring the health benefits of robust social networks (33–38). On the other hand, both social isolation (i.e., living alone or having limited social interactions, termed objective isolation), and loneliness (i.e., subjective distress from a discrepancy between desired and actual social connections), are associated with poorer health and increased risk of early death (39–41).

Yet, differences in social network characteristics by ethnoracial group are also observed, and social disadvantage can affect the extent to which social support serves as a health protector (42, 43). For example, individuals who are White and belong to higher socio-economic groups often have social networks that include professionals such as doctors and lawyers; this might lead to disparities in health outcomes for those from disadvantaged backgrounds for which such ties are absent. It’s important to note, however, that the benefits that should come from higher socio-economic status can be complicated by ethnoracial grouping (44, 45). This is because both subtle and overt forms of discrimination may deter or delay the use of services, despite the apparent network advantages (46). This intertwined relationship between social networks, health, and racial or ethnic inequality (37, 38, 47–49) necessitates a clear understanding of the direct effects of race, ethnicity, and linguistic ability on caregiver health. For some, informal caregiving may heighten the need for social support while also reducing opportunities to engage in social support-building interactions outside of the care dyad, straining preexisting social relationships (19, 35, 50).

Current research comparing multifaceted health outcomes among caregivers of different ethnoracial and linguistic backgrounds remains limited. Thus, in this study, we compare caregivers from three ethnoracial and two linguistic groups. Our aim is to characterize health differences within the caregiving group in NSHAP and highlight ethnoracial and linguistic health disparities for further study. We posit that a holistic examination of health consequences linked with caregiving will enable us to uncover specific vulnerabilities encountered by ethnoracial and linguistic minority caregivers as they age. We expect to observe health disparities within each of five select health domains selected – physical, mental, cognitive, social, and sexual health. To isolate direct effects, we account for network size, an indicator of network strength, as a covariate in our analysis. We hypothesize that ethnoracial and linguistic groupings will have direct effects on health net of social network size. We posit that a comprehensive exploration of health will be crucial for developing targeted support to help these caregivers preserve or improve their health amidst the strains of caregiving.

## Methods

### Data

We sourced data from the first three rounds of NSHAP to examine different health aspects—cognitive, physical, mental, sexual, and social— among informal caregivers. In Round 1 (2005–2006), the sample was constructed using a multistage area probability sampling design with an oversampling of Black and Hispanic adults to generalize to the adult population born between 1920–1947 living in the United States. NSHAP achieved a total sample size of 3,005 individuals with a 75.5% weighted response rate (51). Round 2 (2010–2011) involved re-interviewing the original respondents and surveying their cohabiting spouses/partners, regardless of age (52). In Round 3 (2015–2016), NSHAP revisited surviving respondents from the previous rounds and added a new cohort of adults born 1948–1965, often referred to as the Baby Boomers, along with their cohabiting spouses/partners, regardless of age (53). Additional details about recruitment and sample characteristics can be found elsewhere (52, 54, 55).

All protocols for the collection of human subject data (Protocol Number: 14.06.01) were approved by National Opinion Research Center at the University of Chicago Institutional Review Board (IRB00000967) under its Federalwide Assurance #FWA00000142 and in compliance with U.S. Department of Health and Human Services Office for Human Research Protections regulations. All enrolled participants provided written informed consent under #14.06.01 before being interviewed in the home by trained interviewers. NSHAP data [along with all survey instruments] used for this study are available in the National Archive of Computerized Data on Aging at the Inter-University Consortium of Political and Social Research, https://www.icpsr.umich.edu/web/NACDA/series/706. The details on the development of NSHAP instruments and the implementation of the study design have been published and are also available (56).

### Analytic sample

Caregivers were identified by asking if they were assisting an adult with daily living activities due to aging or disability. They specified their relationship to the care recipient, choosing from spouse, parent, child, grandchild, or other. The proportions of caregivers helping their spouses were 27.5% in Round 1, 38.2% in Round 2, and 28.4% in Round 3. All participants who reported providing care were included in our analysis.

NSHAP’s longitudinal design tracks changes in caregiving status, noting continuity for some and transitions for others across rounds. Given the scarcity of caregivers in the dataset, particularly from diverse ethnoracial and linguistic backgrounds, we selected our sample (N = 1,309) to encompass caregivers from Rounds 1, 2, and 3. We began with 401 caregivers from Round 1, 432 caregivers from Round 2, and 686 caregivers from Round 3. We dropped two cases that were missing data on ethnoracial identity. Next, to specifically focus on the initial caregiving experience reported within NSHAP, we dropped 208 observations from 187 caregivers who reported being a caregiver in a previous round (21 were caregivers across all three rounds and 166 were caregivers in two rounds). In other words, we only used data from their first reported caregiving experience. This approach aims to understand the associations between caregiving and health, while acknowledging the potential differences in outcomes between long-term and short-term caregivers. Consequently, our analysis is based on 1,309 individuals who identified as caregivers in one of the three rounds.

Table 1 provides descriptive statistics of our sample, categorized into five groups based on self-reported ethnoracial identity and language preference based on participants’ chosen language for the NSHAP survey, with English or Spanish as the only options. We used the language in which the survey was administered, from the round at which the respondent first reported being a caregiver. Ethnoracial identity, gender, and age were assessed at baseline (the time of recruitment into the NSHAP survey). Throughout this paper, we use “ethnoracial” to denote identities that combine racial and ethnocultural traits, recognizing these as socially formed and maintained (Bean, 2018; Jiménez et al., 2015). While “Hispanic” isn’t classified as a race, it’s often regarded as one (Bean, 2018). Racial self-identification among Hispanic individuals varies, with many identifying as “White” (65%), others choosing “some other race” (32%), and smaller fractions identifying as “Black” (2.5%), “Asian” (0.2%), or “White, non-Hispanic” (4.6%) (Dietrich & Hernandez, 2022). Due to data constraints, our study does not distinguish between “White Hispanic” and “Black Hispanic” identities. We also didn’t specify cultural heritage but used language preference as a surrogate to explore Hispanic diversity, acknowledging its importance in shaping life experiences and health. Mostly individuals who opted to take the survey in Spanish identified as Hispanic – there were 2 respondents who self-identified as White only and 1 respondent who self-identified as Black and Hispanic who took the survey in Spanish. For the purposes of this study, they were categorized as Hispanic Spanish-speakers. Two Black English-speaking respondents also reported that they were of a Hispanic background – these respondents were coded as Hispanic instead of Black.

### Measures

Our study evaluated caregivers’ health status across several domains—physical, cognitive, mental, social, and sexual health—using a range of measures detailed in Table 2.

#### Physical Health Variables.

We used two main measures of physical health: self-rated health (SRH) and activities of daily living (ADLs). For SRH, respondents rated their overall physical health on a 5-point scale, with values ranging from 1 (poor) to 5 (excellent), where higher scores denote better perceived physical health. For these analyses, we combined the people with poor and fair health together, given the few numbers of people who rated their health as poor, resulting in an adjusted SRH scale from 1–4.

For functional health, respondents described their difficulty levels in performing seven ADLs, such as walking across a room, dressing, and eating. To tailor this measure for caregiving-related functional health, we simplified the ADL assessment. We merged the difficulty categories (combining ‘no difficulty’ with ‘some difficulty’ and ‘much difficulty’ with ‘unable to do’) and omitted certain activities. Specifically, “walking a block” was removed from the ADL scale due to its higher demand compared to other activities, resulting in an adjusted ADL scale from 0 to 6, where higher scores suggest greater functional health challenges.

#### Cognitive Health Variables.

NSHAP changed their cognitive assessment tool between Round 1 and Round 2 because the original measure created a ceiling effect; 96% of Round 1 participants were found to have “normal” cognitive functioning using that measure (57). In Rounds 2 and 3, NSHAP used the survey-adapted Montreal Cognitive Assessment (MoCA-SA), to assess cognitive function across six domains— *orientation, visual spatial, executive functioning, memory, attention, language*, and—on a scale of 0 to 20, where higher scores signify better cognitive abilities (57, 58). For this study of caregivers, we relied solely on global cognition scores and only examined cognition using the MoCA-SA in Rounds 2 and 3.

In addition, to address potential score variations among different ethnoracial and linguistic groups, we included an abbreviated survey-adapted Montreal Cognitive Assessment (MoCA-SAA). The MoCA-SAA aims to reduce measurement variance across diverse groups by focusing on components that are less susceptible to racial bias or errors due to translation. This simplified 4-item tool includes a date-recall task and three elements of the clock-drawing task (59, 60). Designed to provide a concise evaluation of cognitive function, its’ scoring system ranges from 0 to 4. This approach ensures that assessments are more uniformly applicable and reflective of true cognitive ability across different ethnoracial and linguistic populations.

#### Mental Health Variables.

We employed five mental health metrics from NSHAP to assess participants’ emotional well-being: self-rated mental health (SRMH), anxiety, depression, stress, and loneliness. We followed Payne and colleagues’ (2014) detailed instructions on scoring, analyzing, and interpreting the results. SRMH was rated on a 1–5 scale, with higher values indicating better mental health (61). However, like the SRH variable, very few people rated their mental health as “poor.” We combined fair and poor categories, creating a range of 1–4. Anxiety was measured with seven items from the Hospital Anxiety and Depression Scale’s Anxiety Subscale (HADS-A), scored 0–3 based on symptom frequency, leading to a total anxiety score out of 21(62–64). Depression was evaluated using an 11-item Center for Epidemiologic Studies Depression Scale (CES-D) short form (65), with responses indicating symptom frequency over the past week, resulting in a scale of 0–22. Stress was gauged through four Perceived Stress Scale items (66), scored from 0 to 2 to reflect stress frequency, totaling a scale of 0–8. Loneliness was assessed via three items related to the UCLA Loneliness Scale (67, 68), adjusted for consistency across rounds, with scores ranging from 3 to 9 to indicate loneliness levels. These measures, adapted for efficient responses, provide a comprehensive view of participants’ mental health status.

#### Social Health Variables.

To assess the quality of respondents’ social health, we examined variables related to social resources within their social network. We analyzed eight variables that cover support and strain from friends, family, and spouses (69, 70), along with social network size and the proportion of kin (71). Social support was measured by asking respondents about the perceived reliability and openness within their relationships, with scoring adapted for uniformity across survey rounds. We combined similar response categories and calculated mean scores for support from friends, family, and spouses on a scale of 1 to 3, where higher scores indicate greater support. Social strain was evaluated by the frequency of demands and criticism from these groups, using a scoring system that quantifies strain on a scale from 1 to 3, with higher scores indicating more strain.

We used NSHAP’s egocentric social network data to measure the size of the network and the proportion of kin within it. Respondents listed confidants they discuss important matters with, enabling us to count the total number of confidants (0–5) and determine the proportion of kin in their network. For Rounds 2 and 3, we included “grandchild” as a relationship type in calculating the kin proportion, an option that was unavailable in Round 1. We found that even with the addition of “grandchild,” the mean proportion of kin is comparable across the three rounds (see Table 2).

#### Sexual Health Variables.

Depending on the round, about 25–40% of care provided by caregivers was provided to partners. When care was provided to a partner, it is reasonable to anticipate that the dynamics of that relationship would be altered. However, even under circumstances where partners were not care recipients, caregiver strain could reduce relationship happiness and sexual activity. To capture variations in their experiences, we analyzed two key variables: frequency of sex and relationship happiness with partner (70). The frequency of sex was reported by respondents for the past 12 months, with options ranging from “none at all” to “once a day or more.” To maintain consistency across survey rounds, we adjusted the response categories by merging the highest frequencies of sexual activity and introducing a “none” category in Round 1 for those not sexually active with their primary partner in the last year. Relationship happiness was measured on a 1–7 scale, from very unhappy to very happy. In the initial round, participants could rate their happiness with up to two partners, but in later rounds, the focus was solely on the primary partnership. We standardize the assessment of relationship satisfaction across all rounds by considering only the happiness rating with the primary partner in the first round.

### Analytic Plan

We treated physical, cognitive, mental, social, and sexual health as dependent variables with ethnoracial identity and language preference as predictors in separate models. All models included the following covariates: age, gender, and social network size. Ordinary least squares regression was used to explore racial, ethnic, and linguistic disparities in these health dimensions.

Ethnoracial groups, categorized into White, Black, Hispanic, and All Else, was a primary predictor variable. Additionally, the language in which the survey was completed (English or Spanish) was used as another predictor, serving as a proxy for the respondent’s language proficiency. We also included age, and gender (categorized as Man or Woman). Additionally, we incorporated the network size as a covariate in our analysis to isolate the direct effects of ethnoracial and linguistic group membership from the intertwined relationship between social networks and health outcomes highlighted in the literature (37, 38, 47–49). Generally, larger networks are considered more supportive and likely to be diverse, offering a broader range of resources and support. Education level was deliberately excluded from our covariates to focus on health disparities among caregivers who are linguistic minorities, recognizing the high correlations between educational attainment and language proficiency and to avoid multicollinearity. All statistical analyses were conducted using Stata 18 (Stata Corp., College Station, TX.)

## Results

Our study focused on the well-being of caregivers by thoroughly exploring five key health dimensions (physical, cognitive, mental, social, and sexual health) of caregivers. We analyzed data from the National Social Life, Health, and Aging Project (NSHAP) and placed particular emphasis on potential disparities associated with different ethnoracial and linguistic backgrounds. Table 1 provides descriptive statistics of our sample, categorized into five groups based on self-reported ethnoracial identity and language preference.

Our sample consisted of Black English speakers (230 participants, 17.6%), Hispanic English speakers (72 participants, 5.5%), Hispanic Spanish speakers (79 participants, 6.0%), All Else (36 participants, 2.8%), and White English speakers (892 participants, 68.1%). The ‘All Else’ category consisted of individuals not identifying as Black, Hispanic, or White. The gender distribution showed more women in all groups, with the highest female presence among Black caregivers (67%) and the lowest among Hispanic Spanish-speaking caregivers (55.7%).

Educational achievement varied greatly, with a larger proportion of Spanish-speaking Hispanic caregivers (60.8%) having ‘less than high school education,’ contrasting with White caregivers with the lowest proportion (9%). High school/equivalent education levels were fairly uniform among White (26.1%), Black (22.2%), and All Else (22.2%) caregivers; Hispanic caregivers were the exception, with English speakers having the lowest percentage (13.9%) followed by Spanish speakers (15.2%). We found similar levels of ‘some college/associate degrees’ among White (36.9%), Black (40.4%), and English-speaking Hispanic caregivers (38.9%), and lower levels among All Else (25%) and Spanish-speaking Hispanic caregivers (10.1%). All Else (38.9%) and White caregivers (28.0%) had the highest rates of bachelor’s ‘degree or higher,’ while Black (13.5%) and Spanish-speaking Hispanic (13.9%) caregivers had the lowest.

Married was the most common marital status. It was highest among Spanish-speaking Hispanic caregivers (77.2%) and lowest for Black caregivers (55.2%), who also showed higher divorced and widowed statuses. Average ages ranged from 61.9 (All Else) to 68.2 (Whites), with Hispanic and Black caregivers around 65 years; the inclusion of spouses/partners of NSHAP’s older adult respondents widened the full age range, with the youngest and oldest ages being 24 and 92 years, respectively.

To assess the domains of physical, cognitive, mental, social, and sexual health, we employed multiple measures (summation of data provided in Table 2). For physical health, SRH scores suggested that Whites and All Else caregivers reported better health (2.4) compared to Black (2.0) and all Hispanic caregivers (2.0), with English-speaking Hispanics rating their health slightly better (2.1) than their Spanish-speaking counterparts (1.9). Activities of Daily Living (ADLs) scores were uniformly low (0.1) across all groups, indicating minimal disability.

Cognitive health, assessed by the MoCA-SA and MoCA-SAA scales, showed Whites (15.4 for MoCA-SA and 3.4 for MoCA-SAA) and All Else (13.3 for MoCA-SA and 3.4 for MoCA-SAA) outperforming other groups. Spanish-speaking Hispanics scored the lowest (10.4 for MoCA-SA and 3.0 for MoCA-SAA), indicating a disparity in cognitive function across groups.

In terms of mental health, the general trend showed relatively similar scores for SRMH across most groups, but anxiety levels varied, with English-speaking Hispanics exhibiting higher anxiety (6.1) than Spanish-speaking Hispanics (4.6). Depressive symptoms and stress levels showed slight variations. Loneliness scores were relatively uniform across all groups, with ‘All Else’ reporting slightly higher levels (4.5).

Social health dimensions revealed that family and friend support were comparable across groups, with slightly higher family support (2.5) among Spanish-speaking caregivers. However, spouse support was consistent across all groups. Notably, friend and family strain showed minor differences, with the ‘All Else’ category reporting slightly higher strain. Network size and the proportion of kin within one’s network were similar across all groups, indicating no substantial ethnoracial or language-based differences in social network composition.

Sexual health metrics, such as the frequency of sex and relationship happiness, demonstrated minor variations across groups. Relationship happiness scores were slightly lower for English-speaking Hispanics (5.7) and higher for Spanish-speaking Hispanics (6.2).

We compared ethnoracial and linguistic groups with the aim of understanding the differential associations of caregiving on multiple domains of health, and identifying disparate outcomes for further consideration (Tables 3–7). Our analyses centered on individuals who identified as caregivers during any round of NSHAP data collection, exclusively considering data from their initial caregiving report. Due to the small samples among ethnoracial groups, we reported findings that were marginal to traditional levels of significance when p-values were close to the conventional cutoff of p < .05, and we detected patterns across the variables in a given domain that we were able to interpret within a contextual framework (72–75).

### Physical Health

Table 3 presents the association of ethnoracial groups with SRH and functional health, adjusted for age, gender, and social network size. Compared to White caregivers, Black and Hispanic caregivers showed a significant negative association with SRH, (*b* = −0.38, p < 0.001 and *b* = −0.37, p < 0.001), respectively. However, these groups did not significantly differ in their ability to perform daily living activities. The age variable demonstrated a significant negative association with SRH (*b* = −0.01, p < 0.001), indicating a decline in SRH with increasing age, but it showed no significant associations with ADLs. There were no significant associations found between gender and either SRH or ADLs.

### Cognitive Health

Table 4 presents the relationship between ethnoracial groups, age, gender, language, and social network size on cognitive scores. Adjusting for key factors, the analysis uncovered significant disparities in cognitive performance among Black, Hispanic and All else caregivers compared to White caregivers. Specifically, Black caregivers demonstrated markedly lower cognitive scores across all assessments, with a *b* of −2.95 (p < 0.001) for MoCA-SA and *b* of −0.39 (p < 0.001) for MoCA-SAA. Hispanic caregivers also exhibited lower scores than White caregivers on the MoCA-SA (*b* = −2.90, p < 0.001) and MoCA-SAA (*b* = −0.27, p < 0.01). The analysis indicated that Spanish-speaking caregivers scored lower on the MoCA-SA (*b* = −2.20, p < 0.001), but this trend did not extend to the MoCA-SAA where the differences were not significant. This discrepancy for MoCA-SA vs MoCA-SAA was also observed for All Else caregivers (*b* = −2.350, p < 0.001).

Older age consistently correlated with lower scores across the MoCA assessments, suggesting a significant decline in cognitive performance with advancing age: MoCA-SA (*b* = −0.12, p < 0.001) and MoCA-SAA (*b* = −0.01, p < 0.001). In contrast, a larger social network size was associated with higher scores on the MoCA-SA (*b* = 0.37, p < 0.001), with a smaller yet positive effect on MoCA-SAA scores (*b* = 0.03, p = 0.079), slightly above the traditional threshold for significance. Gender differences, however, were not significant across either of the assessments, indicating that cognitive performance disparities were more strongly associated with other demographic and social variables than with gender.

### Mental Health

Table 5 provides a detailed statistical analysis investigating the influence of ethnoracial group, age, gender, language, and social network size on various mental health outcomes among caregivers: SRMH, anxiety, depressive symptoms, perceived stress, and loneliness. Compared to White caregivers, Black caregivers did not exhibit significant associations with most outcomes except for a non-significant trend towards less anxiety (*b* = − .62; p = 0.057) and higher loneliness (*b* = 0.19, p = 0.111). Hispanic caregivers showed marginally significant associations with higher levels of anxiety (*b* = 1.00, p = 0.059), depressive symptoms (*b* = 0.90, p = 0.115), perceived stress (*b* = 0.50, p = 0.105), and loneliness (*b* = 0.30, p = 0.109), though these did not reach conventional significance. Compared with English speaking caregivers, Spanish speakers reported significantly worse SRMH (*b* = −0.38, p < 0.05) and decreased anxiety (*b* = −1.57, p < 0.05); no significant differences in depressive symptoms, perceived stress, or loneliness were noted at conventional significance levels.

Older age had a small but statistically significant negative association with SRMH (*b* = −0.01, p < 0.05) and loneliness (*b* = −0.01, p < 0.05). Gender differences indicated that women experienced significantly worse outcomes than men for SRMH (*b* = −0.12, p < 0.05) and have higher anxiety (*b* = 0.57, p < 0.05), more pronounced depressive symptoms (*b* = 0.83, p < 0.01), and more loneliness (*b* = 0.20, p < 0.05) than men. However, perceived stress did not significantly differ by gender at traditional significance levels.

The impact of social network size on mental health outcomes was minimal, with no significant associations for SRMH, anxiety, depressive symptoms, or perceived stress. However, there was a marginally significant negative association with loneliness (*b* = −0.06, p = 0.051), suggesting a potential protective effect of larger social networks against loneliness.

### Social Health

Table 6 shows associations between demographic variables and measures of social health, specifically focusing on social support, social strain, and network characteristics. It revealed nuanced differences among caregivers based on ethnoracial group and language preference, highlighting how these factors correlate with the size of social networks and perceptions of support and strain within various relationships.

Black caregivers, when compared to White caregivers, reported significantly smaller networks (*b* = −0.39, p < 0.001) and lower levels of support from family (*b* = −0.09, p < 0.05), friends (*b* = −0.12, p < 0.01), and spouses (*b* = −0.12, p = 0.073), and experienced greater strain in relationships with friends (*b* = 0.13, p < 0.001) compared with White caregivers. Although the spouse support finding was marginally above the traditional threshold for significance, it suggested that Black caregivers perceived less support across these relationships compared to their White counterparts.

Hispanic caregivers had a higher portion kin in their networks (*b* = 0.09, p < 0.05), compared with White caregivers, and reported having less support from family (*b* = −0.18, p < 0.05), friends (*b* = −0.30, p < 0.001), and spouses (*b* = −0.18, p = 0.065), and more strain from family (*b* = 0.12, p < 0.05) and friends (*b* = 0.10, p < 0.05). Compared to English-speaking caregivers, Spanish-speaking caregivers reported having more family support (*b* = 0.20, p < 0.05) and less family strain (*b* = −0.19, p < 0.05), with no significant differences in friend or spouse support and strain. All else caregivers had small networks (*b* = −0.43, p = 0.05) experienced less support (*b* = −0.21, p < 0.061) and significantly more strain from friends (*b* = 0.32, p < 0.001).

Age was associated with a significantly larger, kin-centric network (b = 0.01, p < 0.05) that had less friend and spouse support (*b* = −0.01, p < 0.001 and *b* = −0.02, p < 0.001, respectively) but also significantly less strain from family and friends (*b* = −0.01, p < 0.001 and *b* = −0.01, p < 0.001, respectively).

Women had significantly larger networks (*b* = 0.40, p < 0.001), with a greater proportion of kin (*b* = 0.20, p < 0.05), compared to men. They also demonstrated higher levels of family support (*b* = 0.12, p < 0.001) and friend support (*b* = 0.12, p < 0.001) suggesting they feel more supported than men. However, they reported lower spouse support (*b* = −0.09, p < 0.05) and less friend strain (*b* = −0.06, p < 0.01), indicating less support from spouses but also fewer negative interactions with friends. A slight increase in family strain (*b* = 0.07, p < 0.05) was observed, although this was less pronounced compared to family support levels.

### Sexual Health

Table 7 explores the associations between ethnoracial group, age, gender, language, and social network size with two aspects of sexual health: frequency of sex and relationship happiness with their partner. Hispanic caregivers experienced a significantly higher frequency of sex (*b* = 0.34, p < 0.05) but reported lower levels of relationship happiness (*b* = −0.43, p < 0.05) when contrasted with their White counterparts. Caregivers who are Black or of other races did not exhibit statistically significant differences in either the frequency of sex or relationship happiness in comparison. In terms of language, caregivers who speak Spanish, as opposed to English, showed no notable difference in the frequency of sex. However, they did report significantly greater relationship happiness with their partner (*b* = 0.50, p < 0.05).

Age presented a clear negative correlation with the frequency of sex (*b* = −0.05, p < 0.001), indicating that sexual activity tended to decrease as age increased, but it does not have a statistically significant impact on relationship happiness. Gender differences were starkly highlighted, with women reporting a significantly lower frequency of sex (*b* = −0.40, p < 0.001) and reduced relationship happiness (*b* = −0.40, p < 0.001) compared to men. Lastly, the size of one’s social network appeared to have little to no significant impact on both the frequency of sex and relationship happiness, suggesting that the quantity of social connections may not directly influence these aspects of sexual health.

## Discussion

The increasing reliance on caregivers and the disproportionate impact on ethnoracial minorities in the U.S. necessitates a closer examination of caregivers’ health across diverse backgrounds. Unlike most research that typically focuses on one or two health aspects— often physical, mental, or cognitive—the NSHAP dataset, with its comprehensive health measures, enabled us to provide a broader perspective. Our analysis leveraged this extensive set of metrics, spanning physical, cognitive, mental, social, and sexual health, to uncover nuanced health dynamics among caregivers. This approach allowed us to identify disparities and establish a foundation for future research and targeted interventions. Furthermore, our study is pioneering in its examination of caregivers across five distinct health domains, employing the theory of linked health which emphasizes the significance of social and sexual health alongside traditional health measures. To our knowledge, this paper is the first to explore these dimensions collectively to uncover ethnoracial disparities, thereby contributing vital insights into the multifaceted nature of caregiver health.

Significant disparities were found in SRH, with Black and Hispanic caregivers perceiving their health as poorer compared to White caregivers, aligning with existing research on health disparities(16, 76, 77). However, our findings show that these perceived health differences do not translate into significant disparities in functional health, suggesting that caregivers of different backgrounds maintain similar levels of daily functioning.

This discrepancy between perceived and functional health may be due to the study’s focus on community-living caregivers, who are typically able to manage their daily activities effectively. Yet, the issue might also lie with the SRH measure itself (78). While SRH is a widely respected health indicator, its accuracy can be influenced by factors such as educational level, potentially skewing comparisons, especially among less educated groups (79). The observed differences in SRH could reflect real experiences of social disadvantage among Black and Hispanic caregivers, leading them to rate their health more poorly. These perceptions might be exacerbated by the limitations of SRH as a measurement tool. Despite these disparities in health perception, the functional health of caregivers across different groups remains comparable, underscoring the need for community support programs that focus on maintaining and enhancing the physical well-being among caregivers, regardless of their background.

Similarly, our analyses of mental health outcomes were also mixed. In general, we found that being a member of a racial or linguistic minority group was not significantly associated with poorer SRMH among caregivers. However, we did see that Black caregivers exhibited a tendency towards increased depressive symptoms but less anxiety, whereas Hispanic caregivers showed marginally significant links to a range of adverse mental health conditions. Spanish speakers reported significantly poorer SRMH, but less anxiety and loneliness and no differences in depression compared to their English-speaking counterparts. Surprisingly, the size of social networks had limited influence on mental health, except for loneliness, hinting at the need for a more nuanced understanding of these relationships.

We identified clear disparities in social and sexual health, influenced by race, language, and gender. Particularly, compared to White caregivers, Black caregivers experienced lower support and higher strain, and Hispanic caregivers had distinct patterns of social and sexual health, influenced by language preference. Interestingly, Spanish-speaking caregivers reported less strain in their social relationships than their English-speaking counterparts. It seems that while English-speaking Hispanic caregivers have worse social health, Spanish-speaking caregivers enjoyed relatively good social health and by extension, showed better mental health despite having lower ratings of SRMH. It may be the case that friends and family members are more close-knit among linguistic minorities as a way to support each other in a country where they do not speak the dominant language (80).

This trend between English- and Spanish-speaking Hispanics was also observed for sexual health. Hispanic caregivers reported greater sexual activity, but less relationship satisfaction compared to their White counterparts. While Hispanic caregivers (English and Spanish speaking, combined) were less happy in their relationship with their partners than White caregivers, Spanish-speaking Hispanic caregivers were happier in their partnered relationships.

Cognitive health assessments highlighted disparities, with Black and Hispanic caregivers scoring lower than White caregivers. Despite using the MoCA-SAA, a tool designed to minimize racial bias (59), disparities persisted for Black caregivers, underlining the influence of educational, economic, and social factors on cognitive health (81). This pattern is consistent with a recent study that showed marked gender, ethnoracial, and educational disparities in cognitive aging (82). Yet, when the MoCA-SAA was applied to Spanish-speaking Hispanic caregivers, who had the lowest levels of education among all groups, cognitive differences were not evident. This discrepancy may stem from the language-neutral tasks comprising the MoCA-SAA, such as reporting the current date and the clock-drawing task (60). Knowing the month or reading an analog clock tends to be widely understood, although the relevance of reading analog clocks might diminish for future generations with the rise of digital clocks.

The enduring cognitive disparities among Black caregivers underscore the importance of examining the effects of lifelong discrimination (83) and the protective role of quality social relationships. Our study reveals that a larger social network is associated with improved cognitive outcomes, suggesting that social ties may help shield against cognitive decline. However, Black caregivers still report more strain and less support in their social networks compared to White caregivers, linking cognitive health to wider social dynamics. This connection between social support and cognitive health is especially crucial for groups facing systemic social disadvantages, reinforcing the significance of nurturing social connections to address cognitive disparities (33, 34, 37, 38).

While our study sheds light on various health outcomes for caregivers, acknowledging its limitations is crucial for interpretation. A significant limitation of this research is its focus on comparing different groups of caregivers without contrasting these findings with non-caregivers. As a result, the study does not elucidate the specific impacts of caregiving on the analyzed parameters but rather highlights the distinct challenges faced by caregivers across various ethnoracial and linguistic groups. While our study aims to highlight unique challenges and not caregiving’s direct effects, future studies should compare caregivers to non-caregivers to isolate and understand the direct impacts of caregiving. This approach would help determine whether the identified challenges are inherent to caregiving roles or are reflective of broader issues faced by the respective groups, thereby clarifying the actual scope of caregiving-related disparities.

In addition, the reliance on self-reported data could introduce biases such as recall bias or social desirability bias, potentially distorting the results (84, 85). Further, the cross-sectional design, which captures data at a single point, might not fully reflect the caregiving experience’s dynamics or align with critical caregiving phases, limiting our ability to infer causality. Although NSHAP offers longitudinal data, its focus is not specifically on caregiving, possibly restricting the findings’ relevance to broader caregiver populations, especially those in non-community settings or outside the U.S.

This study is also limited by small sample sizes for certain ethnoracial and linguistic groups. These constraints, along with diverse cultural, socio-economic, and healthcare contexts, may prevent a complete understanding of caregivers’ varied experiences and health outcomes. While we adjusted for several key variables, we could not account for all potential factors influencing health disparities, such as the intensity of caregiving responsibilities or the availability of support services. Future research should address these limitations to enhance our understanding of caregiving’s impact on health and inform targeted interventions.

Despite these limitations, our study enriches the understanding of health dynamics among caregivers from diverse backgrounds. We offer two primary contributions to the discourse on the health repercussions of caregiving.

First, we argue for a comprehensive approach to health analysis, extending beyond mere physical health to include social and sexual health —areas frequently overlooked but crucial for understanding overall health outcomes. Our findings underscore the necessity of examining these areas, as they can provide early indicators of health issues before more observable declines in physical, mental, and cognitive health emerge. Particularly, our analysis indicates stark ethnoracial disparities in these less examined domains, revealing significant deficits among Black and Hispanic caregivers. This insight challenges the narrow focus on physical health, which might mask such disparities, and supports the linked health concept that interconnects various health domains, suggesting that struggles in social and sexual health could precede and predict more serious health problems. Moreover, the association between social health, particularly having a purpose in life, and better cognitive outcomes and disability-free longevity, as supported by robust brain science literature (86–88), further validates the importance of considering social health dimensions in promoting healthy aging.

Second, by considering the size of social networks in our analysis, we lay the groundwork for future research to explore how other characteristics of networks (such as their structure, function, and quality) might influence health outcomes (89, 90). This direction also encourages a shift in intervention strategies, suggesting that service providers could achieve better outcomes by focusing on enhancing social network quality, promoting relational happiness, and fostering diverse connections, rather than solely addressing the more apparent physical, mental, and cognitive health issues.

In conclusion, our research underscores the urgency for policy and support systems that comprehensively address caregivers’ multifaceted health needs. It calls for in-depth health evaluations and targeted interventions, emphasizing the need for a broader health perspective that includes, but is not limited to, physical well-being. This approach not only aligns with a more holistic understanding of health but also proposes a shift in focus for service providers towards preventive strategies that enhance social and sexual health, which could ultimately foster better health outcomes across all domains for caregivers.

## Figures and Tables

**Table 1:** Descriptive statistics for sample.

Variables	All Hispanics, N = 151	English-speaking Hispanics, N = 72	Spanish-speaking Hispanics, N = 79	Whites, N = 892	Blacks, N = 230	All Else, N = 36
	%	%	%	%	%	%
**Gender**						
Men	42.4	40.3	44.3	44.0	33.0	41.7
Women	57.6	59.7	55.7	56.1	67.0	58.3
**Education**						
< High School (HS)	47.0	31.9	60.8	9.0	23.9	13.9
HS/Equivalent	14.6	13.9	15.2	26.1	22.2	22.2
Some college/Associate degree	23.8	38.9	10.1	36.9	40.4	25.0
Bachelor’s or more	14.6	15.3	13.9	28.0	13.5	38.9
**Marital Status**						
Married	74.2	70.8	77.2	76.7	55.2	69.4
Living with partner	3.3	1.4	5.1	3.6	4.4	2.8
Separated	2.7	--	5.1	0.1	2.6	-
Divorced	6.0	8.3	3.8	8.0	17.0	19.4
Widowed	8.6	9.7	7.6	8.1	13.5	2.8
Never married	5.3	9.7	1.3	3.6	7.4	5.6
	Mean	Mean	Mean	Mean	Mean	Mean
Age	65.4	65.3	65.6	68.2	65.7	61.9

**Table 2:** Mean Scores for All Health-Related Variables by Ethnoracial/Language Grouping

Domain/Variable	Range	All Hispanics	N	Hispanics, Eng	N	Hispanics, Span	N	Whites	N	Blacks	N	All Else	N
**Physical Health**													
Self-rated (SR) Health	1–4^a^	2.0	151	2.1	72	1.9	79	2.4	890	2.1	230	2.4	36
ADLs	0–6^b^	0.1	151	0.1	72	0.1	79	0.1	892	0.1	230	0.0	36
**Cognitive Health**													
MoCA-SA	0–20^a^	11.6	120	12.7	62	10.4	58	15.4	640	12.6	163	13.3	29
MoCA-SAA	0–4^a^	3.1	120	3.2	62	3.0	58	3.4	641	3.1	163	3.4	29
**Mental Health**													
SR Mental Health	1–4^a^	2.6	149	2.8	72	2.4	77	2.8	871	2.7	213	2.8	33
Anxiety (HADS scale)	0–21^b^	5.3	128	6.1	61	4.6	67	5.1	844	4.5	185	4.6	29
Depressive symptoms (CESD scale)	0–22^b^	5.8	150	6.1	71	5.5	79	5.2	881	5.8	227	5.6	34
Stress (PSS scale)	0–8^b^	2.9	135	3.0	63	2.8	72	2.6	857	2.7	200	2.9	34
Loneliness	3–9^b^	4.3	133	4.4	67	4.2	66	4.1	846	4.4	200	4.5	30
**Social Health**													
Family support	1–3^a^	2.4	149	2.3	71	2.5	78	2.5	872	2.4	222	2.3	36
Friend support	1–3^a^	1.9	146	1.9	71	1.8	75	2.2	876	2.1	225	2.0	36
Spouse support	1–3^a^	2.3	123	2.3	57	2.3	66	2.4	742	2.3	153	2.4	27
Family strain	1–3^b^	1.4	151	1.5	72	1.3	79	1.4	882	1.5	229	1.6	36
Friend strain	1–3^b^	1.2	146	1.2	71	1.2	75	1.1	878	1.3	226	1.5	35
Spouse strain	1–3^b^	1.5	123	1.6	57	1.5	66	1.5	741	1.5	152	1.6	27
Network size	0–5^a^	3.6	148	3.8	70	3.5	78	4.0	887	3.6	227	3.5	35
Proportion kin	0–1^b^	0.6	148	0.6	70	0.6	78	0.5	887	0.5	227	0.5	35
**Sexual Health**													
Frequency of Sex	0–4^a^	1.7	130	1.7	55	1.7	75	1.1	769	1.3	181	1.7	32
Relationship happiness	1–7^a^	6.0	128	5.7	59	6.2	69	6.1	790	5.8	180	5.9	29

a Indicates that higher value represents better outcomes.

b Indicates that higher value represents worse outcomes.

**Table 3:** Association Between Race/Ethnicity, Age, Gender, Language, and Social Network Size with Self-Rated Health (SRH) and Activities of Daily Living (ADLs) in Caregivers

Variables	SRH	ADLs
**Race/ethnicity (v. White)**		
Black	−0.38*** (0.000)	0.01 (0.776)
Hispanic	−0.37*** (0.001)	0.02 (0.649)
All Else	−0.16 (0.313)	−0.06 (0.369)
**Language (v. English)**		
Spanish	−0.11 (0.462)	−0.00 (0.987)
**Age**	−0.01*** (0.000)	0.00 (0.202)
**Gender (v. Men)**		
Women	−0.01 (0.798)	−0.00 (0.873)
**Netwoik size**	0.03 (0.105)	−0.01 (0.120)
N	1295	1297

p-values are in parentheses.

*** p < 0.001;

** p < 0.01;

* p < 0.05

**Table 4:** Associations Between Demographic Factors and Cognitive Performance Scores on the Survey-adapted Montreal Cognitive Assessment (MoCA-SA) and the Abbreviated Survey-adapted Montreal Cognitive Assessment (MoCA-SAA) Among Caregivers.

Variables	MoCA-SA	MoCA-SAA
**Race/ethnicity (v. White)**		
Black	−2.95***(0.000)	−0.39***(0.000)
Hispanic	−2.90***(0.000)	−0.27**(0.006)
All Else	−2.35***(0.000)	−0.11(0.436)
**Language (v. English)**		
Spanish	−2.20***(0.000)	−0.20(0.140)
**Age**	−0.12***(0.000)	−0.01***(0.000)
**Gender (v. Men)**		
Women	0.34(0.108)	−0.00(0.998)
**Network size**	0.37***(0.000)	0.03(0.079)
N	947	948

p-values are in parentheses.

*** p < 0.001;

** p < 0.01;

* p < 0.05

**Table 5:** Associations Between Demographic and Mental Health Indicators (Self-rated Mental Health (SRMH), Anxiety, Depressive Symptoms, Perceived stress, and Loneliness) Among Caregivers.

Variables	SRMH	Anxiety	Depressive Symptoms	Perceived Stress	Loneliness
**Race/ethnicity (v. White)**					
Black	−0.07 (0.301)	−0.62 (0.057)	0.49 (0.162)	0.11 (0.562)	0.19 (0.111)
Hispanic	0.01 (0.939)	1.00 (0.059)	0.90 (0.115)	0.50 (0.105)	0.30 (0.109)
All Else	−0.06 (0.731)	−0.53 (0.480)	0.29 (0.723)	0.50 (0.228)	0.30 (0.272)
**Language (v. English)**					
Spanish	−0.38* (0.014)	−1.57* (0.026)	−0.74 (0.329)	−0.12 (0.775)	−0.26 (0.318)
**Age**	−0.01* (0.033)	−0.01 (0.453)	−0.02 (0.069)	0.01 (0.128)	−0.01* (0.014)
**Gender (v. Men)**					
Women	−0.12* (0.031)	0.57* (0.018)	0.83** (0.002)	0.22 (0.116)	0.20* (0.026)
**Network size**	0.01 (0.588)	0.07 (0.431)	0.04 (0.713)	0.04 (0.397)	−0.06 (0.051)
N	1255	1177	1280	1216	1204

p-values are in parentheses.

*** p < 0.001;

** p < 0.01;

* p < 0.05

**Table 6:** Associations Between Demographic Variables and Social Health Indicators (Family Support, Friend Support, Spouse Support, Family Strain, Friend Strain, Spouse Strain, Network Size, and the Proportion of Kin) Among Caregivers

Variables	Family support	Friend support	Spouse support	Family strain	Friend Strain	Spouse strain	Network size	Proportion Kin
**Race/ethnicity (v. White)**								

Black	−0.09* (0.035)	−0.12* (0.010)	−0.12 (0.073)	0.05 (0.189)	0.13*** (0.000)	−0.00 (0.946)	−0.39*** (0.000)	0.01 (0.809)
Hispanic	−0.18* (0.011)	−0.30*** (0.000)	−0.18 (0.065)	0.12* (0.047)	0.10* (0.021)	0.03 (0.698)	−0.14 (0.387)	0.09* (0.013)
All Else	−0.08 (0.449)	−0.21 (0.061)	−0.09 (0.530)	0.09 (0.285)	0.32*** (0.000)	0.16 (0.166)	−0.43* (0.050)	0.05 (0.372)
**Language (v. English)**								
Spanish	0.20* (0.034)	−0.01 (0.912)	0.05 (0.708)	−0.19* (0.024)	−0.05 (0.367)	−0.09 (0.378)	−0.30 (0.150)	0.02 (0.714)
**Age**	0.00 (0.183)	−0.01** (0.005)	−0.02*** (0.000)	−0.01*** (0.000)	−0.01*** (0.000)	0.00 (0.094)	0.01* (0.021)	0.00*** (0.000)
**Gender (v. Men)**								
Women	0.12*** (0.000)	0.21*** (0.000)	−0.09* (0.047)	0.07* (0.019)	−0.06** (0.002)	−0.03 (0.366)	0.40*** (0.000)	0.12*** (0.000)
N	1267	1273	1040	1286	1275	1038	1297	1297

p-values are in parentheses.

*** p < 0.001;

** p < 0.01;

* p < 0.05

**Table 7:** Associations Between Demographic Factors and Sexual Health: Frequency of Sex and Relationship Happiness

Variables	Frequency of sex	Relationship happiness
**Race/ethnicity (v. White)**		
Black	0.06 (0.563)	−0.19 (0.140)
Hispanic	0.34* (0.036)	−0.43* (0.037)
All Else	0.13 (0.528)	−0.27 (0.353)
**Language (v. English)**		
Spanish	0.11 (0.579)	0.50* (0.029)
**Age**	−0.05*** (0.000)	0.00 (0.837)
**Gender (v. Men)**		
Women	−0.40*** (0.000)	−0.40*** (0.000)
**Network size**	0.02 (0.381)	0.03 (0.351)
N	1104	1118

p-values are in parentheses.

*** p < 0.001;

** p < 0.01;

* p < 0.05

## Data Availability

NSHAP data used for this study are available in the National Archive of Computerized Data on Aging at the Inter-University Consortium of Political and Social Research, https://www.icpsr.umich.edu/web/NACDA/series/706.
